# Corona and Coffee on your commute: A spatial analysis of COVID-19 mortality and commuting flows in England in 2020

**DOI:** 10.1093/eurpub/ckab072

**Published:** 2021-04-19

**Authors:** Igor Francetic, Luke Munford

**Affiliations:** Health Organization, Policy and Economics (HOPE) Group, Centre for Primary Care and Health Services Research, University of Manchester, Oxford Road, Manchester M139PL, United Kingdom

**Keywords:** COVID-19, pandemic, mobility gravity, commuting flows, spatial analysis

## Abstract

**Background:**

The COVID-19 pandemic forced governments to implement lockdown policies to curb the spread of the disease. These policies explicitly encouraged homeworking, hence reducing the number of commuters with the implicit assumption that restricting peoples’ movement reduces risk of infection for travellers and other people in their areas of residence and work. Yet, the spatial interrelation of different areas has been rarely addressed both in the public discourse and in early accounts of the various consequences of COVID-19.

**Methods:**

Our study proposes a spatial analysis of the association between commuting flows and COVID-19 mortality in England between March and June 2020, using a range of publicly available area-level data. To account for spatial correlation, we used a structural mobility gravity model to analyse commuting flows between Local Authority Districts. By accounting for these spatial dependencies, we temper concerns of bias and inefficiency affecting simple linear estimates. Additionally, we disentangle the direct and indirect (from other areas) influence of commuting on COVID-19 mortality.

**Results:**

The results of our spatial regression models suggest that higher commuting flows – in general and particularly by public transport - are associated with higher COVID-19 mortality. Our results are consistent with a reduction in COVID-related mortality after the introduction of a national lockdown in March. The spatial-lag term is statistically significant, highlighting the importance of accounting for spatial dependencies.

**Conclusion:**

We suggest that considering spatial interactions through commuting or travel motivations may offer interesting perspectives on the trade-off between health and economic activity during lockdowns.

**JEL codes**: E65; H75; I18; J61; R23

## 1. INTRODUCTION

During the initial COVID-19 pandemic in 2020, the response of governments across the world was characterized by a widespread use of non-pharmaceutical interventions (1). The strictest form of interventions aimed to restrict physical interactions as much as possible and reduce an individual's freedom of movement (2). These policies often took the form of national and/or regional 'lockdowns', where individuals were instructed to stay in their homes wherever possible. The public health rationale behind lockdowns was the risk of disease spread associated with movement, gathering and mixing of people from different households (3). In economic terms, these restrictions represented an example of a response to the potential negative externalities imposed by individual behaviour on broader society (4). With slight differences in type and timing, lockdowns were implemented almost everywhere across Europe. After initial hesitancy, the UK introduced a first national lockdown on March 23rd 2020 (5), when the Prime Minister Johnson instructed British citizens to "stay at home".

Commuting is the interaction of the housing and labour markets and typically involves employees travelling to and from work. As such, commuting was discouraged during the strictest periods of lockdown in the UK (5) and elsewhere (6), with calls for the adoption of homeworking arrangements for everyone except essential workers.. Commuting to work rather than working from home induces countless opportunities for infection from exposure to environmental factors and the interaction with other co-workers, with the related risk for commuters of transmitting the disease to members of their households and workplaces. A crucial factor exacerbating the risk of infection associated with commuting to and from work is the mode of transport. Private modes of travel (e.g. own car, motorcycle, bicycle or walking) clearly offer less opportunities for contagion compared to public transport aggregating large number of individuals, as suggested by evidence that trains and buses contributed to a large share of infections in the early phases of the pandemic (7). Beyond commuting and intra-national mobility, the international spread of COVID-19 was also assisted by cross-border work (8) and international trade (9).

http://mc.manuscriptcentral.com/ejph

Despite the fact that lockdowns were aimed at restricting movement of people, this specific spatial dimension of infections is often overlooked in the growing body of empirical and theoretical research addressing COVID-19. Notable exceptions to this trend include Birge et al. (10), Glaeser et al. (11) and Fajgelbaum et al. (12). Gravity models provide an interesting theory-grounded approach to account for commuting flows. This concept, which has traditionally been used by economist to study international trade, inspired work by Cuñat and Zymek (13). In a nutshell, the authors derive a structural gravity model for commuting flows based on agents' endogenous choices of work location with given characteristics. This structural gravity model offers a microfounded framework to assess the welfare implications of restrictions to mobility. Furthermore, since commuting flows are a known vector for disease spread, the resulting structural mobility gravity model offers a useful framework to study the spatial spread of disease.

We study the association between commuting and mortality related to COVID-19 between March and June 2020 allowing for spatial correlation across English Local Authority Districts (LADs). LADs are geographical areas with decentralized responsibility for the provision of public services of various types (Appendix A). As of 2020, England is divided into 314 LADs. The spatial interaction between LADs is modelled using weights based on the share of people commuting from one LAD to others (Appendix B).

Our analytical approach tests the following hypotheses:

H1) The higher the percentage of employed people who live in a LAD and work in the ***same*** LAD, the lower the COVID-19 mortality rate.

H2) The higher the percentage of a LAD's workforce who work in a different LAD ***and travel by public transport***, the higher the COVID-19 mortality rate.

H3) The effects tested in H1 and H2 are stronger in the earlier months compared to later months.

http://mc.manuscriptcentral.com/ejph

## 2. METHODS

### 2.1 *Data*

We collated data from a range of publicly available sources. Our main dependent variable is COVID-19 mortality, which is published on a monthly basis at LAD level by the Office for National Statistics (ONS). Specifically, we used age/sex standardised monthly mortality rates (per 100,000 population) for the four-month period from March to June 2020, as well as the monthly mortality rates. Although mortality is an undesirable endpoint of infection and overall disease spread is best represented by reported COVID-19 cases or hospitalizations, these measures are unquestionably related. We focus on COVID-19 mortality for England primarily to overcome the potentially huge heterogeneity in reporting accuracy in reported cases of COVID-19 resulting from wide variability in testing policies across regions and countries in the UK, but also to focus on a more informative measure of the health damage caused on communities by the disease. Notably, the ONS definition for COVID-19 mortality is consistent across areas and time and hence unaffected by differences in testing capacity. In Appendix C4 we also include analyses for overall mortality.

Most other covariates at the level of LAD and for year 2019 (i.e. pre-COVID to avoid reverse causation) were obtained from the ONS through the online portal NOMIS. This includes information on population size, gender distribution, age structure, ethnicity, education, labour force status and mortality rates attributable to respiratory illness from 2019. The ONS also provided the vector data for LAD boundaries used to generate maps. We also obtained population density from the ONS. This indicator encapsulates valuable information about a LAD’s rurality and about living arrangements of its population (i.e. areas with higher density may be assumed to offer more opportunities for infection). The health and disability domain of the Index of Multiple Deprivation (IMD), published by the Ministry of Housing, Communities and Local Government, was obtained from the Government open data portal. Finally, we obtained georeferenced data on care home beds and ratings across England from the Care Quality Commission, which we used to compute LAD level rates of care home beds per person and share of providers with different ratings. No ethical clearance was required for this analysis of secondary publicly available data. A full list of data sources is included in the Appendix C1.

http://mc.manuscriptcentral.com/ejph

We obtained figures on commuting flows between LADs from publicly available 2011 Census data (13). The ONS collected detailed information on location of residence and work, as well as type, frequency and mode of commuting for the whole UK population. Changes in LAD boundaries between 2011 and 2020 resulted in the loss of 14 observations, limiting our analysis to a set of N=298 LADs in England. Our main exposure is the percentage of people who live and work in the same LAD: *HW*. Appendix B2 describes the derivation of *HW* in detail.

The lack of alternative data sources forced us to rely on 2011 Census data on commuting flows, which is arguably old. However, we have no reason to believe that overall commuting patterns have changed radically in the last 9 years.

Figure 1 shows maps describing the distribution of the combined four-month COVID-19 mortality at LAD level and share of people living and working in the same LAD. The maps clearly point to some degree of spatial clustering in the data, but do not reveal a clear-cut relationship.

FIGURE 1 ABOUT HERE

With an analogous approach, we used 2011 Census data on mode of transport for commuting to obtain a variable indicating the share of workers in each LAD commuting to another LAD with public transport. Our definition of "travel by public transport" included bus, train, metro and light rail. Table 1 shows summary statistics for our main outcome, exposure and covariates.

## 2.2 *Empirical approach*

Our study is primarily focused on the relationship between COVID-19 mortality and the extent to which people commute to LADs other than the one where they live, at all and specifically using public transport. Figure 2 shows the bivariate plots for COVID-19 mortality with (a) share of home workers and (b) share of people commuting out with public transport.

FIGURE 2 ABOUT HERE

http://mc.manuscriptcentral.com/ejph

In both cases, the graph seems to suggest the existence of a relationship between commuting and COVID-19 mortality between March and June 2020. On the one hand, a larger share of the workforce living and working in the same LAD is negatively associated with COVID-19 mortality in the same LAD. On the other hand, the share of people living in one LAD and commuting to another by public transport shows a positive association with COVID-19 mortality. However, the simple bivariate relationships above do not account for a range of other factors known to correlate with COVID-19 mortality. As suggested by Figure 1, COVID-19 mortality and commuting flows are likely to entail spatial dependencies, which would invalidate ordinary least squares (OLS) assumptions. Spatial dependencies may be driven by people living and working close in space, or by an economic geography characterized by clustered areas attracting people for work (large urban areas, industrial clusters, etc.) and others where people predominantly live and work in the same area. The existence of spatial correlation is supported by Moran's I test (14), which clearly rejects the null of homoscedasticity at conventional confidence levels, favouring the hypothesis of spatially correlated errors (exact test results are reported in Figure 2's notes). Besides attenuating the risk of bias and inefficiency related to spatial correlation, modelling explicitly the spatial dependencies allows to disentangle direct (own LAD) and indirect (related to changes in other LADs) effects of commuting on COVID-19 mortality (Appendix D).

Our main specification includes spatial lags in mortality rates and in the error terms (15):

$$M_l = \beta C_l + \gamma \boldsymbol{X}_l + \lambda W_{l,k} M_k + u_l; \quad \text{where } u_l = \rho W_{l,k} u_k + \mu_l$$

where subscript $l$ refers to LADs, $M_l$ is COVID-19 mortality (either monthly or four-month mortality between March and June 2020), $C_l$ is the exposure variable of interest related to commuting flows (either $HW_l$ for the percentage of workers living and working in the same LAD or $PT_l$ for the percentage of people commuting for work to a different LAD by public transport) and $X_l$ is a vector of covariates, which we describe in Table 1. The error term $u_l$ includes a spatially correlated component $u_k$ and an orthogonal component $\mu_k$, where $\rho$ models the spatial error autocorrelation structure. The coefficients of interest can be consistently estimated using a generalised two-stage least squares approach.

http://mc.manuscriptcentral.com/ejph

The core of our spatial regression model is the square weight matrix **W**, which gives larger weights to 'nearby' LADs more likely to affect the outcomes of a given LAD $l$. Spatial regression models typically use weight matrices based on contiguity (only neighbouring LADs are assumed to be correlated) or inverse distance (LADs geographically closer get a higher weight). Our approach departs from standard spatial analysis, proposing a weight matrix based on an underlying structural mobility gravity *à la* Cuñat and Zymek (13), such that LADs are related one to another by the share of people commuting between them. A graphical representation, derivation and definition for **W** are provided in Appendix B. **W** satisfies all properties required for a weight matrix in a spatial regression model: it is (semi-)positive definite and has zero on lead diagonals to avoid double weighting of own LAD characteristics (15). Our focus in **W** is on the relative share of commuting flows originating from LAD $l$ and directed to other LADs, irrespective of differences in baseline share of homeworkers in LAD $l$. This should mitigate concerns of endogeneity of the weight matrix, as there is no obvious reason for higher shares of homeworkers to affects the patterns of commuting across nearby LADs, beyond spatial contiguity. It is worth noting that our matrix **W** is not symmetric: the share of people commuting $l \rightarrow k$ is allowed to differ from the share of people commuting $k \rightarrow l$, given that $l \neq k$. Although symmetric spatial weight matrices are often used in (and implied by) applications based on geographical distances, symmetry is not a necessary condition (16). A non-symmetric matrix in this case allows for a more flexible modelling of the spatial interactions in the outcome.

We define the weight matrix based on commuting flows rather than distance for various reasons. Firstly, since people are unlikely to live and work in very distant LADs, commuting flows and (inverse) distance convey at least partially the same information. Secondly, since COVID-19 is transmitted from person to person (rather than through broader geographic or environmental factors), commuting flows seem more relevant to the dynamics of this specific disease compared to arbitrary distance-related weights. Finally, linking back to our motivation in Section 2, this mobility gravity approach offers microeconomic theoretical underpinnings for the spatial interactions observed in the data, potentially allowing welfare evaluations.

http://mc.manuscriptcentral.com/ejph

## 3. RESULTS

### *3.1 Summary statistics*

Table 1 reports the summary statistics for the key variables used. The average four-month COVID-19 mortality rate was 87.5 per 100,000 and this was highest in April (52.9 per 100,000). There was substantial variation in the two commuting variables considered. On average 50% of the employed people who lived in an LAD worked in the same LAD. 17% of those that did commute to another LAD did so by public transport.

On average, 17% of people aged over 16 years of age have no qualifications, although there is a lot of variability in this variable. The male to female ratio is approximately parity.

The unemployment rate pre-COVID varied substantially across England, with some areas having an unemployment rate of 0.9% and some as high as 7.2%. This was linked strongly with deprivation.

Around 17% of the population are aged over 65 years of age, varying from 6.1% to 28.8%. on average, 89% of the population is white, but there is substantial variation here, with a range of 29% to 99%.

2019 mortality rates attributable to respiratory diseases also varied greatly. Generally, LADs with higher mortality rates tended to be in more deprived areas. A similar pattern was observed for the availability and quality of care home beds across England.

TABLE 1 ABOUT HERE

### *3.2 The effect of commuting flows on COVID-19 mortality rates*

In Table 2, we present the results from three forms of Equation (1). The first column is a simple univariate association between the percentage of a LAD's workforce who live and work in the same LAD and the four-month COVID-19 mortality rate in the LAD. The raw association (Figure 2) is -0.824, indicating that a ten percentage point increase in the number of people commuting in and out of another LAD increased COVID-19 mortality by 8.2 deaths per 100,000 ($p<<0.001$). In column (2), we http://mc.manuscriptcentral.com/ejph include additional control variables known to affect the prevalanece of COVID-19. The association remains, but reduces in magnitude as expected. Here, a ten percentage point increase in the number of people commuting in and out of another LAD increased COVID-19 mortality by 6.8 deaths per 100,000 ($p<<0.001$). Notably, the results in columns (1) and (2) are likely to be biased and measured imprecisely due to spatial autocorrelation in COVID-19 mortality across LADs, as suggested visually by Figure 1 and statistically by Moran's I (see exact test results in the notes to Figure 2). Hence, in column (3), we present the results from the model that explicitly accounts for the spatial dependence of COVID-19 spread. Besides a sligthly improved goodness of fit, the spatial model in (3) shows a fairly large (0.64) and strongly significant spatial autocorrelation coefficient. This points to the validity of our spatial approach over OLS. Unfortunately, explicit statistical statistical testing procedures for model selection (OLS vs. spatial, for example the comparison of information criteria) are limited by the generalized two-stages least squares procedure used to estimate our spatial model. The spatial model implies direct and indirect effects of covariates, which can be averaged to obtain average marginal effects (17), as we explain in Appendix D. The direct effect of a ten percent increase in the share of people commuting to other LADs on COVID-19 mortality is an increase of about 2.75 deaths per 100,000 ($p<0.05$). The indirect effect from increases in commuting flows across other LADS is about 4.4 additional deaths per 100,000 ($p<0.05$). The corresponding overall effect is estimated to be around 7.2 deaths per 100,000 ($p<0.05$). More detailed discussion of the estimated coefficients for other covariates are included in Appendix C5, whilst in Appendix C4 we discuss results of similar models for all-cause mortality.

TABLE 2 ABOUT HERE

Additionally, in Appendix C3 we include the results for the full spatial specification (Column (3) of Table 2) but instead consider the monthly mortality rates as the outcome. As expected, and consitent with the idea that lockdown policies reduced the transmition of COVID-19, the main effect is coming through in the March mortality rates. In March there was a very strongly statistically significant relationship between commuting flows and mortality. This effect reduced in magnitude and statistical significance once the lockdown policies were introduced.

http://mc.manuscriptcentral.com/ejph

### 3.3 *The effect of commuting by public transport on COVID-19 mortality rates*

In Appendix C3, we consider the effect of commuting by public transport. Similar to the main effects, there is a very strongly significant association with the March COVID-19 mortality rate. Before lockdown, areas that had higher shares of workers commuting in and out using public transport experienced higher mortality rates. However, this association reduced to close to zero when lockdown policies were implemented.

## 4. CONCLUSION

We found strong evidence to support the idea that areas with higher commuting flows were more susceptible to higher COVID-19 mortality rates. This is most likely due to the fact that there are more people travelling in and out and bringing in the infection from other areas with potentially higher infection rates, either falling ill themselves if vulnerable or spreading the disease in their areas of residence. Our analytical approach builds on a spatial regression model, assuming a weight matrix based on commuting flows between LADs. Overall, we found that a 10 percent increase increases mortality by about 7.2 deaths per 100,000. The decomposition of the effects in the spatial analysis sheds light on the interrelated effects of commuting across LADs: about two thirds of the overall effect of commuting occurs indirectly, through commuting flows and COVID-19 mortality in different LADs. Conversely, only one third can be attributed directly to each LAD own share of commuters.

Our study was not explicitly designed to assess the impact of lockdown policies. However, to some extent, the additional analyses in Appendix C2 support the notion that the lockdown may well have achieved its aim in reducing the severity of the pandemic by reducing the flows of people between geographic areas. We find a strong association between commuting flows for mortality in March, but this effect size reduces to almost zero as we consider later months. However, this assertion relies strongly on the *ceteris paribus* interpretation; i.e. we assume lockdown policies were the only thing that happened in March and there were no other changes in behaviour. Hence, we only make cautious claims about the effectiveness of lockdown policies.

http://mc.manuscriptcentral.com/ejph

In addition to all commuting flows, in Appendix C3 we show that LADs where there was more commuting by public transport experienced stronger mortality. Again, this is consistent with theoretical predictions, in that public transport offers more opportunities for people to be in close contact with their fellow commuters. Private transport, walking and cycling are less likely to see people coming into contact with other people. Like the main effect, the results for public transport also vary by month, with the main effect observed in March, broadly consistent with the notion that the lockdown policy has been effective in curbing the spread of disease and related mortality by limiting interactions between people.

Our study has several limitations. Firstly, we mainly consider COVID-19 mortality as an outcome. The lockdown policies may well have affected other aspects of the lives of workers, such as mental health and employment opportunities, which calls for a broader assessment of the effects of these policies. Further, our measure of commuting flows come from the 2011 Census. No contemporaneous estimates of commuting flows for the whole of England exist. The correlation between commuting flows in 2001 and 2011 was over 0.96, and we have no strong evidence that the changes occurred undermine our findings.

Overall, our work suggests that spatial analyses can be useful to assess COVID risk factors and gauge benefits and costs of lockdown policies. Building on similar approaches to consider commuting flows and other type of motivations to travel, future research may shed light on the unexplored trade-off between health and economic activity intrinsic to lockdown policies. This clearly requires that all relevant consequences of lockdowns beyond COVID-related outcomes – including workers' physical and mental health, costs for increased preventive measures, investments for homeworking arrangements, loss of revenues for firms, etc. - can be effectively measured and considered.

http://mc.manuscriptcentral.com/ejph

**Funding:** Luke Munford is partially funded by the National Institute for Health Research Applied Research Collaboration Greater Manchester [reference: NIHR200174].

**Conflicts of interest:** None declared.

**Authorship statement**: I.F. and L.M. contributed equally to develop the analytical framework, prepare and analyse the data, as well as in writing the manuscript. Both authors approved the final version of the manuscript.

**Data availability statement:** The data that support the findings of this study were obtained from publicly available sources listed in the online Appendix C1.

**Acknowledgments:** Luke Munford is partially funded by the National Institute for Health Research Applied Research Collaboration Greater Manchester [reference: NIHR200174]. The views expressed in this publication are those of the author(s) and not necessarily those of the National Institute for Health Research or the Department of Health and Social Care. The authors are thankful to HOPE seminar participants at the University of Manchester for the useful feedback on earlier versions of the manuscripts.

**Key points:**

- Restricting movement and mixing of people has been among the main motivations for the introduction of extended lockdown and the push towards working from home to curb the COVID-19 pandemic
- We use a spatial structural mobility gravity approach to study the association between commuting flows and COVID-19 mortality in England, between March and June 2020
- We find that a 10% increase in people commuting from one Local Authority District to another is associated with up to 7.2 deaths per 100’000 people, in general and specifically for people commuting with public transport
- Our results suggest that two-thirds of the effect are indirect, i.e. resulting from commuting patterns and COVID-19 mortality from different LADs connected through commuting flows
- The association is strongly reduced after the first national lockdown was introduced in England, providing mild support for the effectiveness of lockdown, although our analysis is not aimed at evaluating lockdown policies and ignores a number of important consequences thereof

**Table 1:** Summary statistics of key variables

| | Obs | Mean | Std. Dev. | Min | Max |
|---|---|---|---|---|---|
| COVID-19 mortality rate (per 100,000 pop.) | | | | | |
| March to June | 298 | 87.54 | 38.08 | 9.50 | 216.60 |
| March | 264 | 9.34 | 9.14 | 1.20 | 47.10 |
| April | 298 | 52.90 | 26.76 | 5.70 | 151.90 |
| May | 295 | 20.51 | 9.36 | 2.00 | 51.30 |
| June | 250 | 6.86 | 4.82 | 0.90 | 36.50 |
| % of employed people in a LAD who live and work in the same LAD | 298 | 49.62 | 16.17 | 15.21 | 90.22 |
| % of people who travel to work in another LAD by public transport | 298 | 17.37 | 16.81 | 2.29 | 76.53 |
| log(Population size) | 298 | 11.92 | 0.54 | 10.59 | 13.95 |
| log(LAD Area, km2) | 298 | 5.31 | 1.19 | 2.48 | 8.09 |
| % of LAD population aged over 16 years of age with no qualifications | 298 | 17.32 | 4.06 | 8.08 | 27.17 |
| Ratio female to males | 298 | 1.03 | 0.03 | 0.88 | 1.10 |
| March 2020 unemployment rate (pre-COVID-19) | 298 | 2.73 | 1.22 | 0.90 | 7.20 |
| | | | | | |
| % of population aged over 65 years of age | 298 | 17.17 | 3.94 | 6.10 | 28.80 |
| % of population who are white | 298 | 88.92 | 13.28 | 29.00 | 98.90 |
| | | | | | |
| Mortality rate due to respiratory diseases in 2019 (per 100,000 pop.) | 298 | 125.21 | 30.66 | 64.17 | 235.53 |
| Care home beds per 10,000 people | 298 | 87.13 | 32.49 | 11.84 | 196.13 |
| % Care Homes rated 'Good' | 298 | 75.65 | 9.11 | 48.00 | 100.00 |
| % Care Homes rated 'Needs improvements' | 298 | 15.63 | 7.56 | 0.00 | 37.04 |
| % Care Homes rated 'Inadequate' | 298 | 1.32 | 2.25 | 0.00 | 18.75 |

Notes: A full list of data sources is available in the Appendix C1.

**Table 2:** The effect of the % of workforce who live and work in the same LAD on the four-month COVID-19 mortality rate

| | (1) Univariate OLS | | (2) Multivariate OLS | | (3) Spatial lag model | |
|---|---|---|---|---|---|---|
| | Coeff. (SE) | *p-value* | Coeff. (SE) | *p-value* | Coeff. (SE) | *p-value* |
| % of employed people in a LAD who live and work in the same LAD | -0.824 (0.128) | <0.001 | -0.676 (0.128) | <0.001 | -0.257 (0.113) | 0.023 |
| Average marginal effects[a] | | | | | | |
| Direct effect | | | | | -0.275 (0.119) | 0.021 |
| Indirect effect | | | | | -0.446 (0.189) | 0.018 |
| Total effect | | | | | -0.722 (0.293) | 0.014 |
| log(Population size) | | | 14.266 (3.569) | <0.001 | 8.277 (3.002) | 0.006 |
| log(LAD Area, km2) | | | 1.171 (1.922) | 0.543 | 0.658 (1.573) | 0.676 |
| % of LAD pop. aged over 16 years of age with no qualifications | | | 1.035 (0.754) | 0.171 | 0.884 (0.622) | 0.155 |
| Ratio female to males | | | 24.500 (61.601) | 0.691 | -50.726 (50.865) | 0.319 |
| March 2020 unemployment rate (pre-COVID-19) | | | 0.749 (2.553) | 0.769 | -0.104 (2.098) | 0.960 |
| % of population aged over 65 years of age | | | -2.821 (0.940) | 0.003 | -1.922 (0.776) | 0.013 |
| % of population who are white | | | -0.981 (0.198) | <0.001 | -0.893 (0.163) | <0.001 |
| Health domain of Index of Multiple Deprivation (Base=1 'best') | | | | | | |
| 2nd quintile | | | 3.160 (5.395) | 0.559 | -0.339 (4.426) | 0.939 |
| 3rd quintile | | | 7.605 (6.601) | 0.250 | 3.060 (5.424) | 0.573 |
| 4th quintile | | | 8.712 (8.085) | 0.282 | -0.252 (6.682) | 0.970 |
| 5th quintile (= 'worst') | | | 6.443 (9.319) | 0.490 | -3.048 (7.699) | 0.692 |
| Mortality rate due to respiratory diseases in 2019 | | | 0.330 (0.079) | <0.001 | 0.174 (0.067) | 0.009 |
| Care home beds per 10,000 people | | | 0.041 (0.069) | 0.552 | 0.120 (0.057) | 0.034 |
| %CH rated 'Good'[b] | | | 0.422 (0.280) | 0.133 | 0.131 (0.230) | 0.570 |
| %CH rated 'Needs improvements'[b] | | | 0.007 (0.320) | 0.983 | -0.195 (0.262) | 0.458 |
| %CH rated 'Inadequate'[b] | | | 0.475 (0.705) | 0.501 | 0.039 (0.576) | 0.946 |
| Constant | 128.438 (6.690) | >0.001 | 47.329 (70.425) | 0.502 | 47.294 (58.498) | 0.419 |
| Spatial lags[c] | | | | | | |
| Outcome (λ) | - | - | - | - | 0.644 (0.067) | <0.001 |
| Error term (ρ) | - | - | - | - | -0.005 (0.121) | 0.966 |
| Observations | 298 | | 298 | | 298 | |

Notes: [a] The definition of average marginal effect (direct, indirect and total) for the spatial model is provided in Appendix D. [b] The reference level of Care Home quality rating is "Very good". [c] The weight matrix S used in the spatial regression model is defined in section 2.1; it links LADs based on commuting flows between LADs reporting the share of people commuting to each LAD out of all people commuting out of a the home LAD (i.e. where they live). The matrix S has zeros on the leading diagonal and is asymmetric (i.e. the share of people commuting from A to B are allowed to be different from that of people commuting from B to A). Standard errors in parentheses.

**Figure legends**

**Figure 1**: Spatial patterns in COVID-19 mortality and the % of employed people in a LAD who live and work in the same LAD

**Figure 2**: Associations between COVID-19 mortality and (a) % of employed people in a LAD who live and work in the same LAD and (b) % of people who commute out of an LAD by public transport

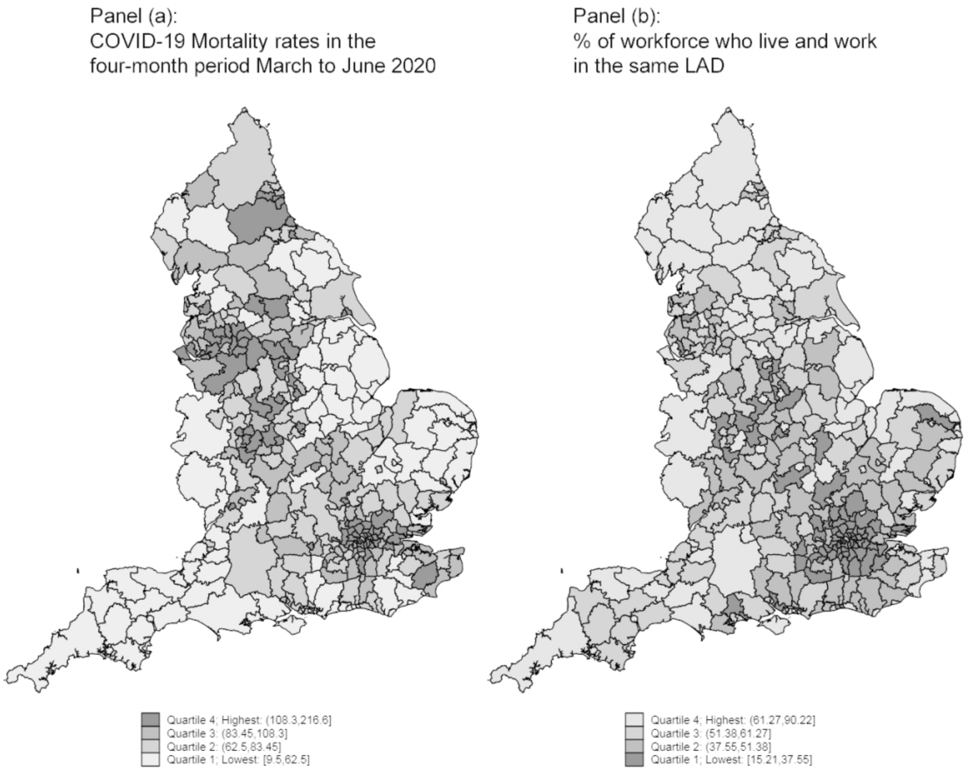

Spatial patterns in COVID-19 mortality and the % of employed people in a LAD who live and work in the same LAD

136x114mm (250 x 250 DPI)

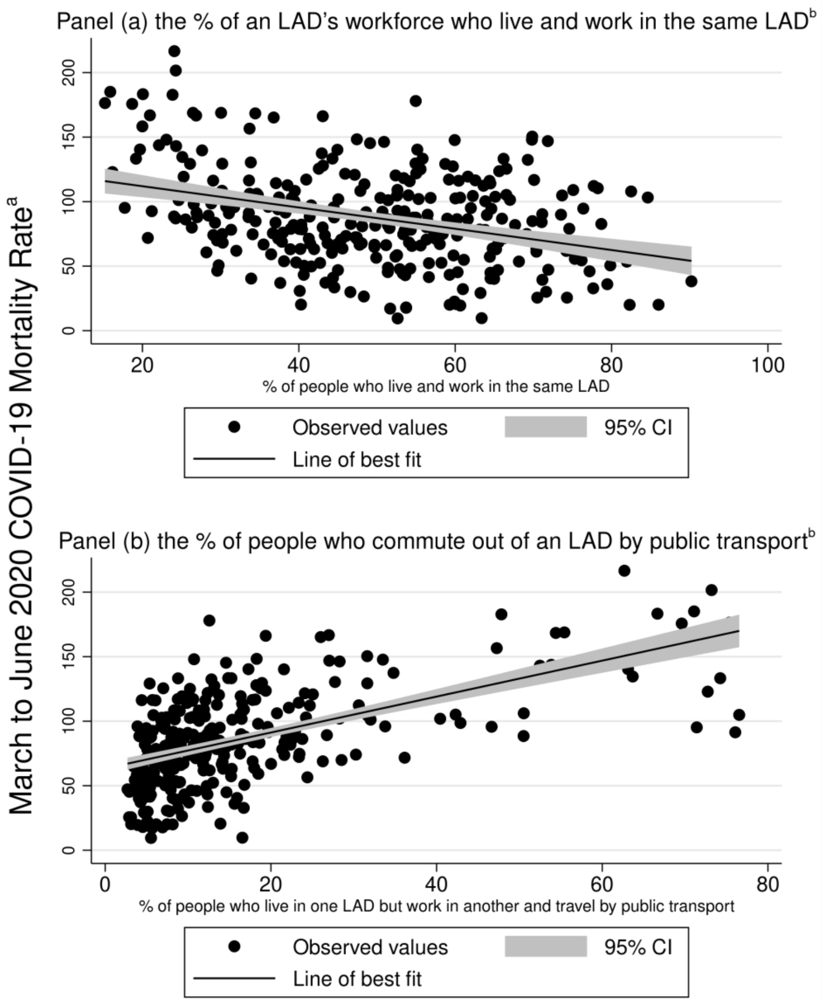

[a] Mortality rates are per 100,000 population, and adjusted for age and sex. [b] We applied the Stata routine based on Moran's I test to assess the existence of spatial correlation in the regression residuals. Under the null of no spatial correlation in the residuals and assuming the spatial weight matrix W defined in Appendix B, the resulting test statistics are distributed as $\chi^2(1)$. The value of Moran's I test statistics for the residuals of the simple regressions above are 144.33 ($p<0.000$) for panel (a), and 237.96 ($p<0.000$) for panel (b) respectively. Hence, Moran's I tests suggest the rejection of the null of homoscedasticity, hence pointing towards the existence of substantial spatial correlation in the residuals.

Associations between COVID-19 mortality and (a) % of employed people in a LAD who live and work in the same LAD and (b) % of people who commute out of an LAD by public transport

177x254mm (300 x 300 DPI)
