# Supplementary material for: Corona and coffee on your commute: a spatial analysis of COVID-19 mortality and commuting flows in England in 2020
Source: Eur J Public Health. 2021 Apr 19;31(4):901–7. doi: 10.1093/eurpub/ckab072 (PMC8083223; doi:10.1093/eurpub/ckab072)
Supplement: ckab072_Supplementary_Data [file ckab072_supplementary_data.docx]

*Corona and Coffee on your commute:
A spatial analysis of COVID-19 mortality and commuting flows in England in 2020*

Igor Francetic^1🖂^, Luke Munford^1^

Version: March 2021

^1^ Health Organization, Policy and Economics (HOPE) Group, Centre for Primary Care and Health Services Research, University of Manchester, Oxford Road, Manchester M139PL, United Kingdom.

🖂 Corresponding author: Igor Francetic, Email: igor.francetic@manchester.ac.uk

**Online Appendix**

**Appendix A:** Description and structure of Local Governments in England

Local government in England operates either under a one- or two-tier system. The one tier system is comprised of 152 unitary authorities (or Upper-tier Local Authorities) whereas the two-tier system if made up of 314 county and district councils (or Local Authority Districts (LADs), or Lower-tier Local Authorities). LADs are the lowest level of local government. The distinction between Upper-tier and LADs is that many Upper-tier local authorities re broken down into several districts. However, some Upper-tiers and LADs are perfectly co-terminus.

As of 2020, there are 314 LADs in England, including 36 metropolitan boroughs, 32 London boroughs, 188 non-metropolitan districts and 56 unitary authorities (perfectly co-terminus), as well as the City of London and Isles of Scilly which are also districts, but do not correspond to any of these categories. In our analyses, we exclude the City of London and the Isles of Scilly due to their unique characteristics (low populations). The 32 London Boroughs also have an ‘umbrella’ organisation, or a combined authority; the Greater London Assembly.

The primary aim of LADs is to provide subnational, local, government. We provide a brief overview of the responsibilities of each type of LAD IN Table D1, below.

Metropolitan boroughs typically have populations of between 175,000 and 1,100,000. London Boroughs typically have populations of between 150,000 and 300,000. Non-metropolitan district typically have populations of between 50,000 and 250,000. Unitary authorities typically have populations of between 40,000 and 550,000.

**Table A1:** Key responsibilities of each type of Local Authority District

| Service | Non-metropolitan  county | Non-metropolitan  district | Unitary  authority | Metropolitan  borough | London  boroughs | Greater London  Assembly |
| --- | --- | --- | --- | --- | --- | --- |
| Education | X |  | X | X | X |  |
| Housing |  | X | X | X | X | X |
| Planning applications |  | X | X | X | X |  |
| Strategic planning | X |  | X | X | X | X |
| Transport planning | X |  | X | X | X | X |
| Passenger transport | X |  | X | X |  | X |
| Highways | X |  | X | X | X | X |
| Fire | X |  | X | X |  | X |
| Social services | X |  | X | X | X |  |
| Libraries | X |  | X | X | X |  |
| Leisure and recreation |  | X | X | X | X |  |
| Waste collection |  | X | X | X | X |  |
| Waste disposal | X |  | X | X | X |  |
| Environmental health |  | X | X | X | X |  |
| Revenue (taxation) collection |  | X | X | X | X |  |

*Source: material is sourced from* [*https://www.local.gov.uk/about/what-local-government*](https://www.local.gov.uk/about/what-local-government) *and* [*https://www.politics.co.uk/reference/local-government-structure/*](https://www.politics.co.uk/reference/local-government-structure/)

**Appendix B**

**Appendix B1**

**Figure B1**: Conceptual representation of alternative weight matrix options for spatial regression models

| *Panel (a): Lattice representing hypothetical weights in a weight matrix based on contiguity. Only areas neighbouring to L are weighted positively.* |
| --- |
| 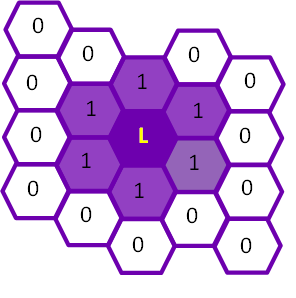 |
| *Panel (b): Lattice representing hypothetical weights in a weight matrix based on inverse distance. Weights decrease moving away from L.* |
| 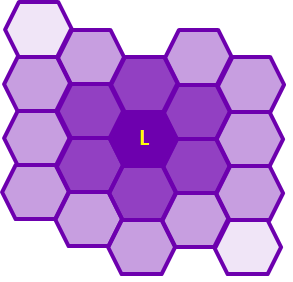 |
| *Panel (c): Lattice representing hypothetical weights in a weight matrix based on commuting flows. Weights depend upon the number of people commuting between L and other areas (the arrows).* |
| 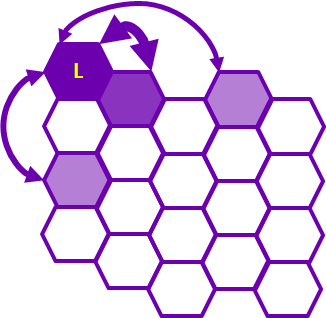 |

**Appendix B2:** Derivation of weight matrix W and main exposure HW

We started from data on commuting flows for a set of N=298 LADs in England. We compiled these data on commuting flows to form a square $N\times N$ matrix **A**. The element $a_{i,j}$ in matrix **A** represents the number of people living in LAD *i* and commuting for work to LAD *j*:

$$\mathbf{A}=\left( \begin{matrix} a_{1,1} & \cdots& a_{1,N} \\ \vdots& \ddots& \vdots\\ a_{N,1} & \cdots& a_{N,N} \end{matrix} \right)$$

We used the diagonal component of matrix **A** to obtain the share of people living and working in the same LAD, i.e. homeworkers (*HW*). Element $i$ in this new vector *HW* is defined as

$${HW}_{i}=\frac{a_{i=j}}{\sum_{j=1}^{N} a_{i,j}} \forall i,j\in\{1,2,\ldots,N\}$$

Starting from **A,** we then imposed zeroes on the leading diagonal and applied a row-normalization to obtain the weight matrix **W**:

$$\mathbf{W}=\left( \begin{matrix} 0 & w_{1,2} & \ldots& w_{1,N} \\ w_{2,1} & 0 & \ldots& w_{2,N} \\ \vdots& \vdots& \ddots& \vdots\\ w_{N,1} & w_{N,2} & \ldots& 0 \end{matrix} \right)$$

Linking back to the notation in **A,** element $w_{i,j}$ represents the share of commuting workers living in LAD *i* commuting to LAD *j*:

$$w_{i,j}=\frac{a_{i,j}}{\sum_{j=1}^{N} a_{i,j}}\forall i\neq j\in\{1,2,\ldots,N\}$$

Imposing zeroes on the leading diagonal ensures that we only focus on people commuting out of their LAD of residence. The row-normalisation ensures that each row sums to 1, or $\sum_{j=1}^{N} w_{i,j}=1$.

The interpretation of these manipulations is that our weight matrix **W** only focuses on how commuting flows originating from LAD *i* are relatively spread across of other LADs, irrespective of differences in baseline share of homeworkers in LAD *i*.

**Appendix C**

**Appendix C1**

**Table C1:** Data sources for all variables

| Variable | Reference year | Source |
| --- | --- | --- |
| COVID-19 mortality rate (per 100,000 pop.) | 2020 | Office for National Statistics, COVID-19 specific mortality data, LAD level |
| Commuting flows between English LADs | 2011 | Office for National Statistics, Census 2011, Data on commuting patterns |
| % of people commuting by public transport | 2011 | Office for National Statistics, Census 2011, Data on commuting patterns |
| LAD Area, km2 | 2019 | Office for National Statistics, Open Geography Portal, LAD boundaries elaborated with QGIS software |
| Population size | 2019 | Office for National Statistics, Nomis portal |
| % of LAD population aged over 16 years of age with no qualifications | 2019 | Office for National Statistics, Nomis portal |
| Ratio female to males | 2019 | Office for National Statistics, Nomis portal |
| March 2020 unemployment rate (pre-COVID-19) | 2020 | Office for National Statistics, Nomis portal |
| % of population aged over 65 years of age | 2019 | Office for National Statistics, Nomis portal |
| % of population who are white | 2019 | Office for National Statistics, Nomis portal |
| Mortality rate due to respiratory diseases in 2019  (per 100,000 pop.) | 2019 | Office for National Statistics, Nomis portal |
| Care home beds per 10,000 people | 2019 | Care Quality Commission, Care Home data |
| %CH rated 'Good' | 2019 | Care Quality Commission, Care Home data |
| %CH rated 'Needs improvements' | 2019 | Care Quality Commission, Care Home data |
| %CH rated 'Inadequate' | 2019 | Care Quality Commission, Care Home data |

**Appendix C2**

**Table C2:** The effect of the % of workforce who live and work in the same LAD on the four-month and monthly COVID-19 mortality rate

|  | (1)  March to June | | (2)  March | | (3)  April | | (4)  May | | (5)  June | | |
| --- | --- | --- | --- | --- | --- | --- | --- | --- | --- | --- | --- |
|  | Coeff. (SE) | *p* | Coeff. (SE) | *p* | Coeff. (SE) | *p* | Coeff. (SE) | *p* | Coeff. (SE) | *p* | |
| % of employed people in a LAD who live and work in the same LAD | -0.257 (0.113) | 0.023 | -0.128 (0.025) | p<0.001 | -0.083 (0.083) | 0.318 | -0.001 (0.039) | 0.980 | 0.000 (0.024) | 0.999 | |
|  |  |  |  |  |  |  |  |  |  |  | |
| Average marginal effects ^a^ |  |  |  |  |  |  |  |  |  |  | |
| Direct effect | -0.275 (0.119) | 0.021 | -0.133 (0.026) | <0.001 | -0.091 (0.090) | 0.313 | -0.001 (0.042) | 0.980 | 0.000 (0.030) | 0.999 | |
| Indirect effect | -0.446 (0.189) | 0.018 | -0.134 (0.038) | <0.001 | -0.180 (0.166) | 0.278 | -0.002 (0.068) | 0.980 | 0.000 (0.162) | 0.999 | |
| Total effect | -0.722 (0.293) | (0.293 | -0.268 (0.055) | <0.001 | -0.271 (0.252) | 0.284 | -0.003 (0.110) | 0.980 | 0.000 (0.192) | 0.999 | |
|  |  |  |  |  |  |  |  |  |  |  | |
| Other controls ^b^ | Yes |  | Yes |  | Yes |  | Yes |  | Yes |  | |
| Spatial lags |  |  |  |  |  |  |  |  |  |  | |
| Outcome (λ) | 0.644 (0.067) | <0.001 | 0.522 (0.068) | <0.001 | 0.692 (0.075) | <0.001 | 0.646 (0.075) | <0.001 | 0.873 (0.117) | <0.001 | |
|  |  |  |  |  |  |  |  |  |  |  | |
| Error term (ρ) | -0.005 (0.121) | 0.966 | 0.074 (0.150) | 0.623 | -0.073 (0.118) | 0.537 | -0.310 (0.135) | 0.022 | -0.462 (0.239) | 0.053 | |
| Observations | 298 | | 264 | | 298 | | 295 | | 250 | | |
| Notes: ^a^ The definition of average marginal effect (direct, indirect and total) for the spatial model is provided in Appendix C  ^b^ Other controls are as listed in Table 2. Full output is available on request. Standard errors in parentheses. | | | | | | | | | | |  |

**Appendix C3**

**Table C3:** The effect of the percentage of workforce who commute out of an LAD using public transport on COVID-19 mortality rates

|  | (1)  March to June | | (2)  March | | (3)  April | | (4)  May | | (5)  June | | |
| --- | --- | --- | --- | --- | --- | --- | --- | --- | --- | --- | --- |
|  |  |  |  |  |  |  |  |  |  | | |
|  | Coeff. (SE) | *p* | Coeff. (SE) | *p* | Coeff. (SE) | *p* | Coeff. (SE) | *p* | Coeff. (SE) | *p* | |
| % of people who travel to work in another LAD  by public transport | 0.179 (0.132) | 0.177 | 0.142 (0.038) | <0.001 | 0.105 (0.099) | 0.285 | -0.004 (0.045) | 0.933 | -0.020 (0.027) | 0.468 | |
|  |  |  |  |  |  |  |  |  |  |  | |
| Average marginal effects ^a^ |  |  |  |  |  |  |  |  |  |  | |
| Direct effect | 0.195 (0.144) | 0.175 | 0.146 (0.038) | <0.001 | 0.116 (0.108) | 0.283 | -0.004 (0.049) | 0.933 | -0.026 (0.035) | 0.458 | |
| Indirect effect | 0.405 (0.303) | 0.181 | 0.108 (0.029) | 0.001 | 0.260 (0.241) | 0.281 | -0.007 (0.085) | 0.933 | -0.258 (0.509) | 0.612 | |
| Total effect | 0.600 (0.440) | 0.172 | 0.253 (0.053) | <0.001 | 0.276 (0.344) | 0.275 | -0.011 (0.133) | 0.933 | -0.284 (0.531) | 0.593 | |
|  |  |  |  |  |  |  |  |  |  |  | |
| Other controls ^b^ | Yes |  | Yes |  | Yes |  | Yes |  | Yes |  | |
| Spatial lags |  |  |  |  |  |  |  |  |  |  | |
| Outcome (λ) | 0.702 (0.062) | <0.001 | 0.439 (0.087) | <0.001 | 0.719 (0.070) | <0.001 | 0.663 (0.078) | <0.001 | 0.930 (0.113) | <0.001 | |
|  |  |  |  |  |  |  |  |  |  |  | |
| Error term (ρ) | -0.069 (0.114) | 0.545 | 0.054 (0.143) | 0.707 | -0.076 (0.113) | 0.499 | -0.329 (0.136) | 0.015 | -0.565 (0.239 | 0.018 | |
| Observations | 298 | | 264 | | 298 | | 295 | | 250 | | |
| Notes: ^a^ The definition of average marginal effect (direct, indirect and total) for the spatial model is provided in Appendix C  ^b^ Other controls are as listed in Table 2. Full output is available on request. Standard errors in parentheses. | | | | | | | | | | |  |

**Appendix C4**

**Table C4:** The effect of the % of workforce who live and work in the same LAD on the four-month and monthly all-cause-19 mortality rate

|  | (1)  March to June | | (2)  March | | (3)  April | | (4)  May | | (5)  June | | |
| --- | --- | --- | --- | --- | --- | --- | --- | --- | --- | --- | --- |
|  |  |  |  |  |  |  |  |  |  | | |
|  | Coeff. (SE) | *p* | Coeff. (SE) | *p* | Coeff. (SE) | *p* | Coeff. (SE) | *p* | Coeff. (SE) | *p* | |
| % of employed people in a LAD who live and work in the same LAD | -0.185 (0.177) | 0.297 | -0.154 (0.063) | 0.014 | 0.178 (0.117) | 0.879 | 0.041 (0.059) | 0.482 | 0.095 (0.048) | 0.049 | |
|  |  |  |  |  |  |  |  |  |  |  | |
| Average marginal effects ^a^ |  |  |  |  |  |  |  |  |  |  | |
| Direct effect | -0.187 (0.179) | 0.296 | -0.154 (0.063) | 0.014 | 0.187 (0.123) | 0.879 | 0.043 (0.061) | 0.482 | 0.097 (0.049) | 0.049 | |
| Indirect effect | -0.065 (0.059) | 0.269 | -0.012 (0.017) | 0.455 | 0.021 (0.141) | 0.881 | 0.332 (0.049) | 0.499 | 0.043 (0.029) | 0.137 | |
| Total effect | -0.251 0.235) | 0.285 | -0.166 (0.062) | 0.008 | 0.040 (0.263) | 0.880 | 0.076 (0.109) | 0.488 | 0.140 (0.075) | 0.064 | |
|  |  |  |  |  |  |  |  |  |  |  | |
| Other controls ^b^ | Yes |  | Yes |  | Yes |  | Yes |  | Yes |  | |
| Spatial lags |  |  |  |  |  |  |  |  |  |  | |
| Outcome (λ) | 0.264 (0.057) | <0.001 | 0.075 (0.102) | 0.464 | 0.552 (0.075) | <0.001 | 0.455 (0.058) | <0.001 | 0.317 (0.080) | <0.001 | |
|  |  |  |  |  |  |  |  |  |  |  | |
| Error term (ρ) | 0.192 (0.115) | 0.096 | 0.183 (0.150) | 0.223 | -0.012 (0.126) | 0.922 | -0.348 (0.134) | 0.009 | -0.169 (0.0166) | 0.306 | |
| Observations | 298 | | 298 | | 298 | | 298 | | 298 | | |
| Notes: ^a^ The definition of average marginal effect (direct, indirect and total) for the spatial model is provided in Appendix C  ^b^ Other controls are as listed in Table 2. Full output is available on request. Standard errors in parentheses. | | | | | | | | | | |  |

Unlike COVID-19 mortality, there is much less of an association between the main exposure and all-cause mortality rates. This is consient with the main message of this paper that commuting flows can affect the spread of highly contagious – particualrly respiratory – conditions, but does not have much of an effect on other causes or mortality, particualrly non-communicable diseases (NCDs).

**Appendix C5:** Comments on coefficients for control variables

On the whole, the other covariates behave as expected. LADs with larger population experienced higher mortality rates. LADs with a higher proportion of white people also experienced lower mortality rates, consistent with the epidemiological evidence that COVID-19 can effect BAME communites more severely. LADs with higher pre-COVID respiratory mortality rates also experienced higher mortality rate, consistent with the idea that more vulnerable people were more likely to be affected. We do observe, however, that LADs with higher proportions of their population who are aged 65 years and above have lower mortality rates. This seems counterintuitive, as there is strong evidence to suggest – at the individual level – that older people were more susceptible. However, there may be a counteracting ‘healthy survivor’ argument, in that LADs with a higher proportion of over 65s are more likely to have higher life expectancy and hence better levels of health.

**Appendix D:** Interpretation of regression estimates for direct, indirect and total effects

Our spatial models assume the existence of spatial dependencies in the COVID-19 mortality. Specifically, we model this spatial dependence as a function of the share of people commuting for work to a LAD different from where they live.

This does not only affect estimation but also the interpretation of the estimated coefficients. The vector of coefficients in a standard OLS settings represent average partial effects of the regressor (i.e. the share of commuters) on the dependent variable (i.e. COVID-19 mortality), among independent observations. However, allowing for spatial correlation implies that observation are not independent anymore: the level of COVID-19 mortality in LAD *i* is affected by neighbouring LADs which have commuters commuting flows in common with *i.* Interpreting the coefficients requires taking this into account.

Following LeSage (2008) and LeSage&Thomas-Agnan (2015) – besides reporting the vectors of estimated coefficients - we present the results using the following summary measures to convey the average effects measured in our analyses.

**Average Direct Effect:** This is the average across all LADs in the sample of the (“own”) effect of marginal changes in share of commuters in LAD *i* on the COVID-19 mortality in the same LAD *i*, including the potential feedback effect from neighbouring LADs which are affected by changes in LAD *i’s* COVID-19 mortality (again through some common commuting flows).

**Average Indirect Effect:** This is the average across all LADs in the sample of the marginal effect on COVID-19 mortality for LAD *i* resulting ONLY from changes in commuting flows across other LADs ≠ *i*, indirectly affecting COVID-19 mortality in LAD *i* as a result of spatial dependencies through shared commuting flows.

The **Average Total Effect** is just the sum of the two above (**Average Direct Effect+ Average Indirect Effect**).

**References:**

LeSage JP, An Introduction to Spatial Econometrics, Revue d'économie industrielle. 2008:123:19-44.

LeSage JP, Thomas‐Agnan C. Interpreting Spatial Econometric Origin-Destination Flow Models. Journal of Regional Science. 2015;55(2):188–208
